# A Molecular Dynamics Analysis of the Thickness and Adhesion Characteristics of the Quasi-Liquid Layer at the Asphalt–Ice Interface

**DOI:** 10.3390/ma17061375

**Published:** 2024-03-17

**Authors:** Yunhao Jiao, Yujin Yao, Heping Qiu, Huaxin Chen, Yongchang Wu

**Affiliations:** School of Materials Science and Engineering, Chang’an University, Xi’an 710061, China; yunhaojiao13@163.com (Y.J.); yujinyao@chd.edu.cn (Y.Y.); hepingqiu@chd.edu.cn (H.Q.); hxchen@chd.edu.cn (H.C.)

**Keywords:** asphalt, ice adhesion, quasi-liquid layer, molecular dynamics simulation

## Abstract

The quasi-liquid layer (QLL), a microstructure located between ice and an adhering substrate, is critical in generating capillary pressure, which in turn influences ice adhesion behavior. This study employed molecular dynamics (MD) methods to obtain QLL thickness and utilized these measurements to estimate the adhesive strength between ice and asphalt. The research involved constructing an ice–QLL–asphalt MD model, encompassing four asphalt types and five temperature ranges from 250 K to 270 K. The QLL thickness was determined for various asphalts and temperatures using the tetrahedral order parameter gradient. Additionally, capillary pressure was calculated based on the QLL thickness and other geometric parameters obtained from the MD analysis. These findings were then compared with ice adhesion strength data acquired from pull-off tests. The results indicate that QLL thickness varies with different asphalt types and increases with temperature. At a constant temperature, the QLL thickness decreases in the order of the basal plane, primary prism plane, and secondary prism plane. Furthermore, the adhesion strength of the QLL diminishes as the temperature rises, attributed to the disruption of hydrogen bonds at lower temperatures. The greater the polarity of the asphalt’s interface molecules, the stronger the adhesion strength and binding free energy. The MD simulations of the asphalt–ice interface offer insights into the atomic-scale adhesive properties of this interface, contributing to the enhancement in QLL property prediction and calibration at larger scales.

## 1. Introduction

In winter, the formation of a smooth ice layer on asphalt roads significantly endangers traffic safety by reducing skid resistance and creating potential hazards. Various anti-icing strategies have been developed to prevent ice accumulation on roads, primarily focusing on the physical and chemical interactions between the asphalt surface and ice. Concurrently, research has identified the presence of a quasi-liquid layer at the interface between asphalt and ice. Insights from the QLL [1] theory can offer valuable understanding and inform effective solutions.

The adhesive strength of the QLL has been demonstrated to have a correlation with its thickness [2]. Previous studies have primarily investigated the thickness and structure of the QLL on ice surfaces using experimental methods such as X-ray Diffraction (XRD) [3], Atomic Force Microscopy (AFM) [4,5], and Transmission Electron Microscopy (TEM) [6,7], among others [8,9]. These approaches have indicated that the QLL thickness ranges from 1 to 45 nm. However, these methods have yet to establish a successful link between QLL thickness and premelting temperatures. Meanwhile, molecular dynamics simulations have directly linked QLL thickness with temperature, becoming increasingly effective in providing detailed insights into the dynamic behavior of molecules at the microscopic level [10,11,12]. Through simulation studies, Conda [13] calculated the QLL thickness at different temperatures. Donadio elucidated the thickness of the QLL layer at the ice–air interface near melting points and the effect of NaCl adsorption on QLL thickness [14]. Concurrently, simulations exploring the QLL thickness and its structure at interfaces between ice and other substances are emerging. Zhang [15] first accurately measured the thickness of the QLL using the dynamic properties of water molecules, assessing the changes in QLL thickness at different temperatures. Anderson measured the QLL’s thickness at the ice–silicon interface, observing it to be approximately 1 nm on silicon at 263 K [16]. Shota conducted simulations on the structure of silica dioxide and its interfacial QLL layer, analyzing its density distribution and structural details [17]. This article investigates the relationship between the thickness of the QLL formed at the asphalt–ice interface and its adhesive properties through MD simulations.

Investigating the atomic interactions between asphalt and water molecules offers a more effective method for analyzing the interface between asphalt road surfaces and ice. The creation of an accurate asphalt model is thus of paramount importance. In simulating asphalt, it is essential to establish equilibrium in geometrical structures and total energies, which involves determining bond lengths, bond angles, and atomic charges [18,19]. The Hildebrand parameter [20] facilitates the identification of structural functional groups. Murgich [21] calculated asphalt’s lowest energy structure. Li and Greenfield [22] developed a 12-component model for three asphalt types. Hansen et al. [23] created a four-component molecular model, followed by a dynamic relaxation analysis. Greenfield [22,24] subsequently refined the 12-component model to better reflect authentic asphalt properties, highlighting the correlation between chemical and mechanical properties within the asphalt model. MD simulations also shed light on atomic interactions at the asphalt interface. Wang and Wu [25,26] found that water molecules influence the adhesive strength of the asphalt–aggregate interface. The adhesion between asphalt mixtures and ice, as well as the impact of salts on the asphalt–ice interface under extreme environmental conditions, continues to be explored in depth [27,28]. Zhao [29,30] studied the impact of road surface characteristics on the adhesion between ice and concrete. Luo [31] focused on the adhesive strength relationships at the asphalt–aggregate interface, particularly the anisotropy of mineral surfaces in the aggregate. Zou’s research delved into the impact of extreme saline environments.

**Figure 1:**
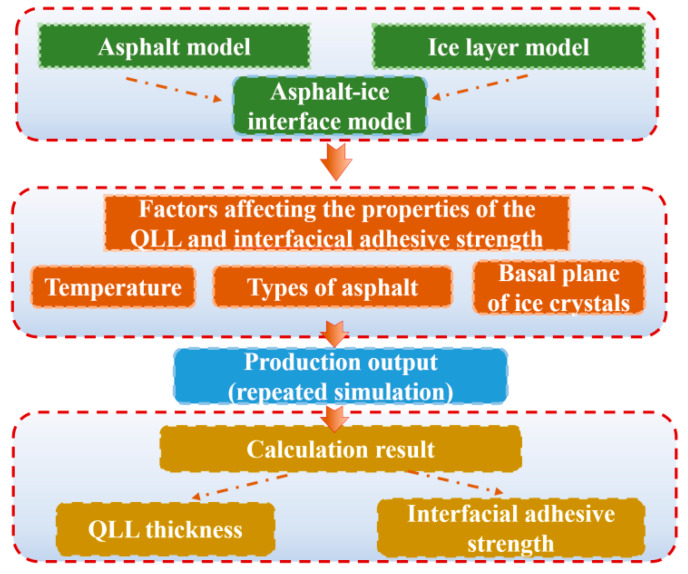
Research objectives’ flowchart.

In prior studies, Chen [32] developed a quasi-liquid layer (QLL) freeze-adhesion strength model, integrating theory and empirical data, which yielded commendable results. Wu [32,33] established a correlation between the freeze-adhesion strength, contact angle, and freeze-adhesion temperature using the QLL sandwich model and capillary pressures. Qiu [34,35,36] combined QLL and microwave technology for de-icing concrete pavements. However, research specifically targeting the QLL at the ice–asphalt interface has been limited. A thorough investigation of QLL thickness and the characteristics of the freeze-adhesive interface remains to be conducted. Additionally, primary studies have predominantly focused on macroscopic experiments, lacking a detailed molecular analysis. Addressing these gaps, this paper explores the molecular dynamics of the QLL at the ice–asphalt interface, examining its thickness, structure, and adhesive strength. The study simulates the QLL across various asphalt models at different temperatures, calculates the QLL’s thickness in three planes, analyzes the pattern of thickness variation with temperature changes, and further examines factors influencing this variation. The goal is to understand the adhesive mechanism of the QLL at the ice–asphalt interface, which involves determining the interfacial tension, contact angle, and adhesion strength.The specific flowchart is shown in Figure 1.

## 2. Simulation Methodology

### 2.1. Analysis of Material Models

#### 2.1.1. Asphalt Model 

In our study, we utilized four distinct asphalt models. The first model is tri-component asphalt derived from a comprehensive atomistic approach. The remaining three models, developed and refined by Greenfield and Li [24], include the AAA-1, AAA-K, and AAA-M asphalt models. These are composed of twelve molecular components; three molecules represent asphaltenes, two molecules represent Saturates, two molecules represent naphthene aromatics (DOCHN and PHPN), and five molecules represent polar aromatics. They offer a more precise representation of varying asphalt components. The calculated densities and thermal expansion coefficients of these models are in close agreement with experimental findings [37], thereby enhancing the depiction of asphalt polymers’ dynamic properties. Details of each molecule’s characteristics are presented in Table 1, while the composition of each asphalt model is detailed in Table 2.

#### 2.1.2. Water and Ice Models

**Figure 2:**
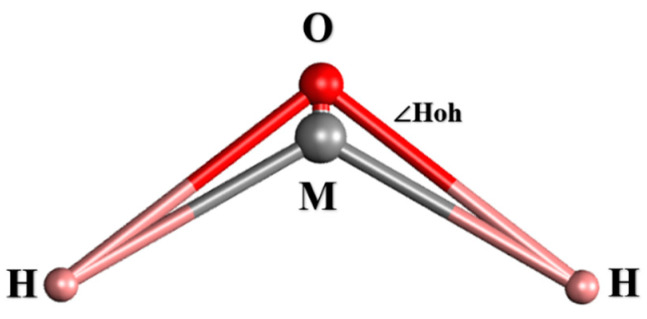
Schema of the TIP4P water model (point M is a virtual point).

Water molecules were represented using the TIP4P-ICE water model [38]. This model, part of the TIP4P series, is renowned for its extensive use in MD simulations due to its ability to accurately mimic water’s phase diagram and efficiency in large-scale simulations. It is particularly favored for simulating the water–ice interface, given its precise rendering of ice lattice structures and melting temperatures. The basic parameters of the TIP4P water model are listed in Table 3, and the schematic diagram of the TIP4P-ICE water model is shown in Figure 2.

**Figure 3:**
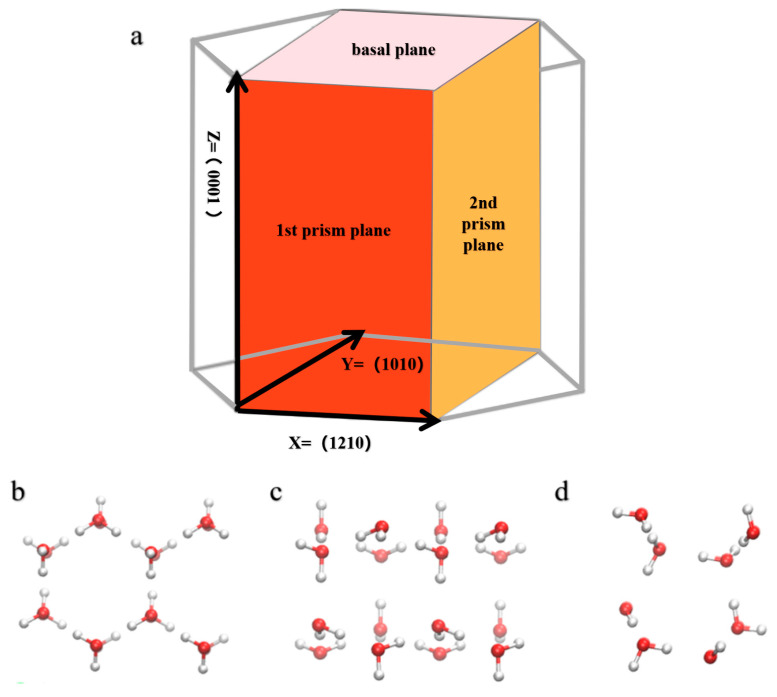
(**a**) The three most prominent planes, basal plane (0001), primary prismatic plane (1010), and secondary prismatic plane (1120), viewed from the top of the unit cell model. (**b**) Basal plane, (**c**) primary prismatic plane, and (**d**) secondary prismatic plane. Red atoms represent oxygen, while white atoms represent hydrogen.

Hexagonal ice (Ih) utilizing the TIP4P-ICE model was selected [39], due to its relatively simple structure and suitability for MD simulations. Figure 3a illustrates the three prominent planes of the ice (Ih) crystal structure: the basal plane (0001), the primary prismatic plane (1010), and the secondary prismatic plane (1120) [40,41]. These planes exhibit distinct structural and property differences, which are depicted in the top views shown in Figure 3b–d for each respective plane. The density (ρlh) of ice crystal Ih was calculated to be 0.906 g/cm^3^, which is close to the experimental value of 0.917 g/cm^3^. The simulated premelting temperature (Tm) of 272.2 K is also near the experimental data of 273.15 K, and the calculated enthalpy change (H_Ih_) = 14.60 kcal/mol is nearly consistent with experimental results [37,42]. Therefore, using the ice crystal Ih structure composed of TIP4P-ICE can adequately ensure the simulation’s realism.

#### 2.1.3. MD Simulation Details

The initial energy-minimized states of each molecule were obtained using Gaussian-16. The molecular models were constructed with Packmol, and MD simulations were conducted using GROMACS 2020.3 software. The subsequent visualization of the simulation results was performed with VMD 1.9.3 software. In the GROMACS [43] simulations, the OPLS-AA force field [44,45] was employed, and Equations (1)–(5) define the various potential energy parameters in the force field. It is known for its compatibility with the TIP4P water model and suitability for simulating organic polymer molecules.

(1) $$E_{r^{N}}=E_{bonds}+E_{angles}+E_{dihedrals}+E_{bondeded}$$

(2) $$E_{bonds}=\sum_{bonds}K_{r}{\left(r-r_{0}\right)}^{2}$$

(3) $$E_{angles}=\sum_{angles}k_{\theta }{\left(\theta -\theta_{0}\right)}^{2}$$

(4) $$E_{diherdrals}=\sum_{diherdrals}\left\{\begin{array}{c} \frac{V_{1}}{2}\left[1+\cos\left(\emptyset -\emptyset_{1}\right)\right]+\frac{V_{2}}{2}\left[1+\cos\left(2\emptyset -\emptyset_{2}\right)\right]\\ +\frac{V_{3}}{2}\left[1+\cos\left(3\emptyset -\emptyset_{3}\right)\right]+\frac{V_{4}}{2}\left[1+\cos\left(4\emptyset -\emptyset_{4}\right)\right] \end{array}\right\}$$

(5) $$E_{nonbonded}=\sum_{i>j}\frac{A_{ij}}{r_{ij}^{12}}-\frac{C_{ij}}{r_{ij}^{6}}+\frac{q_{i}q_{j}e^{2}}{4\pi \varepsilon_{0}r_{ij}}$$

Bond lengths and angles were modeled as harmonic oscillators, and non-bonded interactions were represented by the Lennard-Jones potential (see Equation (6)). Additionally, a cutoff radius of 1 nm was set [46].

(6) $$V_{LJ}=4\varepsilon \left[{\left(\frac{\sigma }{r}\right)}^{12}-{\left(\frac{\sigma }{r}\right)}^{6}\right]A$$

In the establishment of the asphalt model, long-range electrostatic interactions were calculated using the Particle Mesh Ewald (PME) method [47] and cutoff method. Settings were applied as follows: rvdw = 1 nm, rlist = 1 nm, and rcoulomb = 1 nm, with the Fourier Transform (FFT) grid spacing set at 0.15 nm. These settings ensured that adequate simulation precision was achieved while maintaining controlled computational costs, and the cutoff radius was set to 1 nm. When the interface was simulated, the LINCS [48] constraint algorithm maintained constant bond lengths, adjusting them as necessary. Temperature-coupled simulations utilized the Nosé–Hoover [49] thermostat with a time constant of 0.5 ps. For pressure-coupled simulations, the Parrinello–Rahman barostat [50] was used. Atomic trajectories were computed using the Verlet [51] algorithm. Periodic boundary conditions and None-Periodic boundary conditions (wall in *z*-axis) were applied during the simulation. Regarding the use of None-Periodic boundary conditions (wall in *z*-axis), we selected a 9-3 Lennard-Jones wall to simulate the fixed effects of the interface, while designating C and O atoms as the constituent atoms of the wall. We manually disabled the rlist feature to avoid computational errors caused by volume issues, while opting for the cutoff method to calculate the electrostatic potential energy. The Nosé–Hoover thermostat and Parrinello–Rahman barostat were utilized to control temperature and pressure.

### 2.2. Ice–QLL–Asphalt

#### 2.2.1. Asphalt Model Verification and Optimization

**Figure 4:**
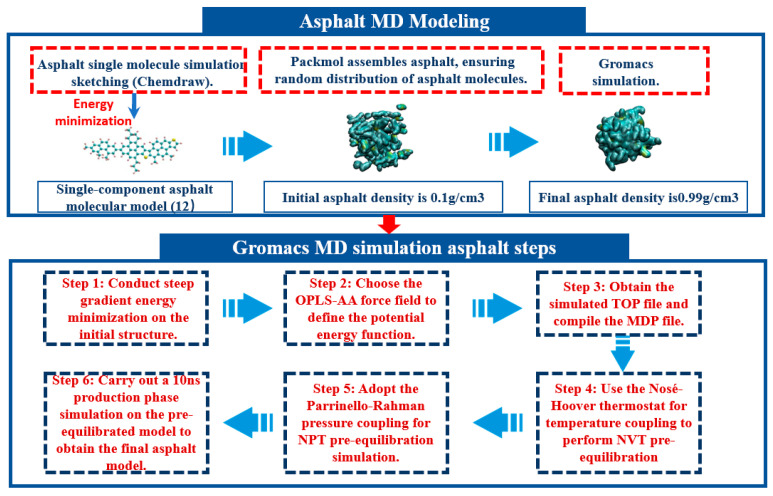
Molecular dynamics simulation workflow for the asphalt model.

The entire procedure of the asphalt model simulation is depicted in Figure 4. Initially, the asphalt model was derived from a 10 ns production run in the NPT ensemble, utilizing Nosé–Hoover temperature coupling and Parrinello–Rahman pressure coupling methods. Subsequent calculations of density, viscosity, and glass transition temperature for the production phase asphalt model were performed. These calculated values were then compared with actual test values, as presented in Table 4. This comparison aimed to validate the simulated asphalt model’s accuracy and reliability.

#### 2.2.2. Ice–QLL–Asphalt Model

**Figure 5:**
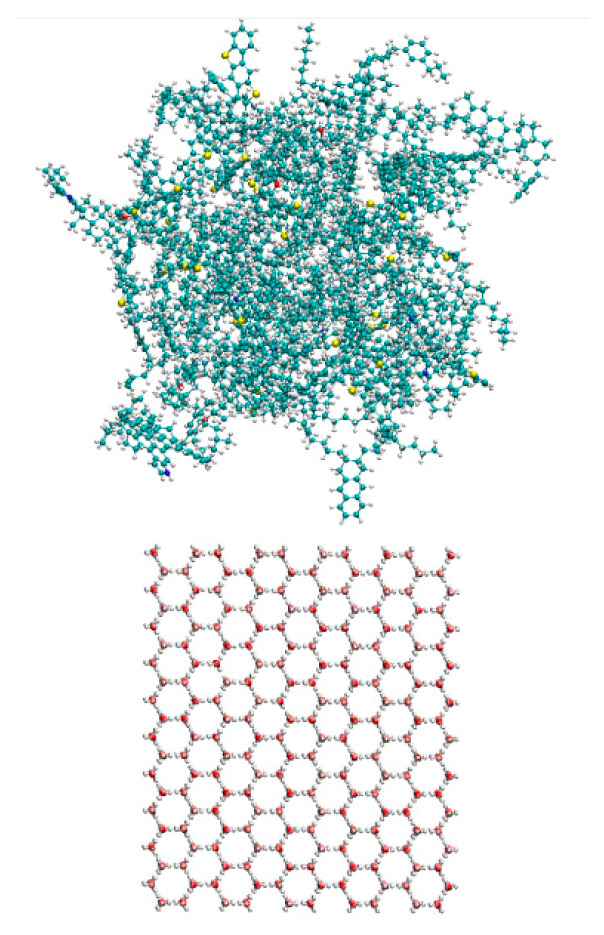
Diagram of the asphalt–ice interface model.

We constructed a 50 Å × 40 Å × 55 Å hexagonal ice crystal (Ih) using 2000 TIP4P-ICE water molecule models [52]. The asphalt model was then placed 0.5 nm from the ice surface to prevent molecular overlap [53]. The asphalt and ice crystal interface model is depicted in Figure 5.

Initially, we performed energy minimization on the interface model. This was followed by a 500 ps NPT ensemble simulation using Berendsen temperature and pressure coupling, aiming to achieve a pre-equilibrated phase of the interface. A subsequent 10 ns production phase simulation at 200 K was conducted for two boundary conditions and each temperature gradient, employing the NPT ensemble with Nosé–Hoover temperature coupling and Parrinello–Rahman pressure coupling, to finalize the production phase interface model. Observations at lower temperatures indicated atomic distributions consistent with a water–ice mixture layer at the interface. The analysis of density variations along the *z*-axis and the relative concentration of oxygen atoms helped identify this layer as the QLL. Therefore, the resulting model is appropriately termed the ice–QLL–asphalt model.

## 3. Simulation Task

### 3.1. QLL Thickness Task

The thickness of the QLL by simulation represents a significant methodological approach in studying this layer. A key objective in measuring the thickness of the condensed layer is to differentiate between liquid water and solid ice. Given the frequent updates and revisions in the definition of hydrogen bonds [54], this paper employs the tetrahedral order parameter concept, as proposed by Errington and Debenedetti [55], to distinguish between these two states. The parameter has proven to be extremely effective in characterizing the structure of vitreous water [56]. The definition of the tetrahedral order parameter *q_i_* is provided in Equation (7).

(7) $$q_{i}=1-\frac{3}{8}\sum_{j=1}^{3}\sum_{k=j+1}^{4}{\left(\cos\left(\theta_{i,j,k}\right)+\frac{1}{3}\right)}^{2}$$

where the sum is over the four nearest neighbors (oxygens) of the oxygen of the *i*th water molecule. The angles *i*, *j*, and *k* are the angles formed by the oxygens of molecules *i*, *j*, and *k* (molecule *i* being the vertex of the angle). The tetrahedral order parameter adopts a value of 1.

Equation (8) is obtained by defining the probability density P(q).

(8) $$P(q)=<\frac{N(q)}{\left(N\Delta q\right)}>$$

where *N* is the number of molecules in a given configuration having an order parameter between *q* and *q* + Δq, with Δq being the size of the grid. The brackets in the above equation mean an ensemble average.

Further, from the probability density, Equation (9) can be derived.

(9) $$1=\int P\left(q\right)dq$$

Then, a threshold value, *q_t_*, is established based on the probability density *p*(*q*). Due to the overlap exhibited in the probability density graph, $q_{t}$ is obtained through the integration of equality. A molecule is classified as being in an ice state if its *q* value exceeds *q_t_*; otherwise, it is in a liquid water state. This criterion is formalized in Equation (10).

(10) $$\int_{q_{t}}^{1}P_{liquid}\left(q\right)dq=\int_{0}^{q_{t}}P_{Ih}\left(q\right)dq$$

$\int_{q_{t}}^{1}P_{liquid}\left(q\right)dq$ represents the probability of incorrectly labeling a liquid-like state as ice-like, and $\int_{0}^{q_{t}}P_{Ih}\left(q\right)dq$ represents the probability of incorrectly labeling an ice-like state as liquid-like. Equation (10) incorporates the probability of incorrectly classifying a liquid-like state as ice-like and the probability of misclassifying an ice-like state as liquid-like. By equating the probabilities of incorrect assignments, the errors cancel out. For the TIP4P-ICE model, the threshold *q_t_* is set at 0.9. If the *q* value is less than *q_t_* = 0.9, the molecule is identified as liquid water. Conversely, if it exceeds this threshold, the molecule is categorized as solid ice.

### 3.2. Adhesion Strength Task

#### 3.2.1. Surface Tension and Contact Angle

The values of surface tension and interfacial tension directly influence the adhesive characteristics of the interface between two materials, both of which are related to intermolecular forces. Based on Irving and Kirkwood’s method, the surface and interfacial tensions are calculated from the microscopic pressure tensor, as shown in Equation (11).

(11) $$\gamma =\frac{1}{2}\left[P_{ZZ}-\frac{1}{2}\left(P_{XX}+P_{YY}\right)\right]A$$

where *γ* is interfacial tension or surface tension. *P_XX_*, *P_YY_*, and *P_ZZ_* denote the pressure tensors in their respective X, Y, and Z directions. Additionally, A signifies the contact area between two materials within the interfacial tension framework.

The area is calculated using the Monte Carlo method; the core lies in random sampling, which involves projecting a large number of random points and then determining the proportion of these points that fall within the range of the contact interface. From this proportion, the interface area A can be calculated. In this study, if the interfacial asphalt completely covers the ice surface, the interface area can be considered equal to the area of the ice layer. The unit of area is Å^2^.

In the asphalt–QLL–ice system, there exist two contact angles. There is a certain relationship between the contact angles and the interfacial and surface tensions. The contact angle can be calculated using Young’s equation, as presented in Equations (12) and (13). The contact angle reflects the interfacial free energy of the materials. It plays a crucial role in subsequent calculations of interface adhesive strength.

(12) $$cos\theta_{1}=\frac{\left(\gamma_{1}-\gamma_{2}\right)}{\gamma_{5}}$$

(13) $$cos\theta_{2}=\frac{\left(\gamma_{3}-\gamma_{4}\right)}{\gamma_{5}}$$

where *cosθ*_1_ and *cosθ*_2_ represent the contact angles for the ice–liquid and asphalt–liquid layers, respectively. The γ_1_ refers to the surface tension between ice and air, while γ_2_ indicates the interfacial tension between the ice and liquid layer. Similarly, γ_3_ corresponds to the surface tension between asphalt and air, γ_4_ to the interfacial tension between the asphalt and liquid layer, and γ_5_ to the surface tension of the liquid layer.

#### 3.2.2. Interface Adhesion Strength

At the interface between ice and asphalt, an adhesive condensed layer was formed. To calculate the adhesive strength of this layer, we simplified the ice-condensed-layer–asphalt system into a capillary adhesion model, resembling a liquid-filled gap between two solid plates, as depicted in the figure. In this scenario, capillary-induced pressure [57] emerges at the interface’s ends, promoting adhesion between the two plates. A model was developed by utilizing the Laplace equation, by Cai and Bhushan [58], to estimate this capillary-induced pressure between two plates with differing surface properties. The equation determining the pressure exerted on the surface of the condensed layer is outlined in Equation (14).

(14) $$P=\frac{2\gamma \left(cos\theta_{1}+cos\theta_{2}\right)}{H}$$

where the *θ_i_* represents the angle between the liquid layer meniscus and the ice layer, while *θ_s_* indicates the angle between the liquid layer meniscus and the substrate, *H* denotes the thickness of the liquid layer, and γ signifies the surface tension of the liquid layer.

#### 3.2.3. Binding Free Energy

The binding free energy in this study is used to indicate the intermolecular interaction energy between the ice and asphalt interface molecules. The MM-PBSA method is employed to calculate the binding free energy between the molecules at the ice–asphalt interface. The system’s free energy is represented by the following equations, Equations (15)–(17):

(15) $$G=E_{MM}-TS_{conf}$$

(16) $$E_{MM}=E_{bonded}+E_{elec}+E_{vdw}$$

(17) $$G=E_{bonded}+E_{elec}+E_{vdw}-TS_{conf}$$

where E_MM_ represents the average molecular potential energy in a vacuum, which is obtained by MD simulation. E_bonded_ includes interactions such as the bond length, bond angle, and dihedral angle, E_elec_ is the electrostatic energy, and E_vdw_ is the van der Waals energy. TSconf represents the contribution of entropy to the free energy.

Then, we can derive the binding free energy of the interface, as shown in Equation (18).

(18) $${\Delta G}_{bind}=G_{complex}-G_{asphalit}-G_{ice}$$

where G_complex_ represents the free energy of the QLL layer. G_ice_ represents the free energy of ice lh. In addition, G_asphalt_ represents the free energy of the asphalt molecules.

## 4. Results and Discussion

### 4.1. Investigation of QLL Thickness at the Asphalt–Ice Interface

**Figure 6:**
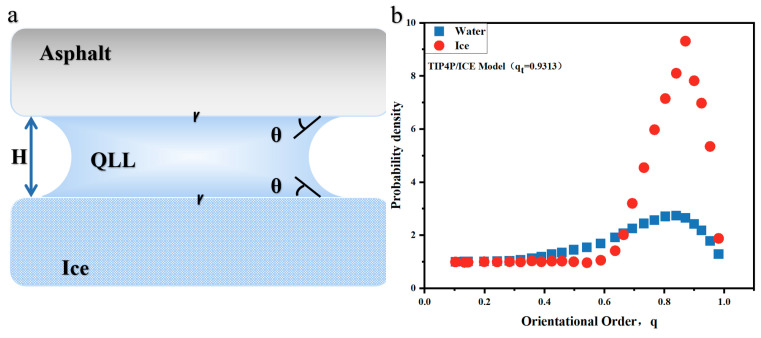
(**a**) Simplified sandwich model of asphalt–QLL–ice. (**b**) Density plot of the tetrahedral order parameter for ice and water.

In the molecular dynamics simulation of the asphalt–ice contact interface, we observed the spontaneous formation of an amorphous ice layer at the interface, referred to as the QLL in this paper. Figure 6a shows the simplified ‘sandwich’ model of the asphalt–QLL–ice system. Simultaneously, we calculated and illustrated the probability density of ice and water in Figure 6b. Using a predefined threshold, molecules with values exceeding 0.9 were categorized as ice-like, while those below this threshold were identified as water-like. The aim of this study is to calculate and examine the thickness variations of the interface’s condensed layer from diverse perspectives, to identify the factors that influence its thickness.

#### 4.1.1. Impact of Crystal Facets of Ice on the Thickness of the QLL

**Figure 7:**
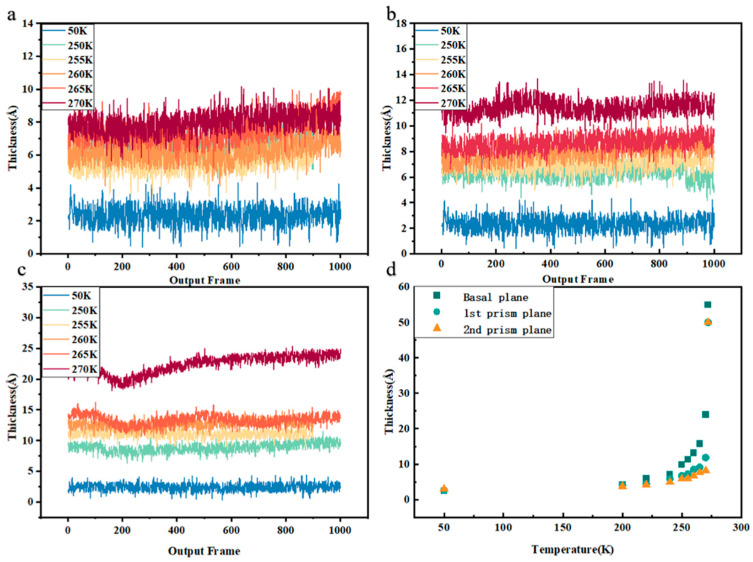
(**a**–**c**) represent instantaneous QLL thickness on the basal plane, primary plane, and secondary plane at different temperatures (blue—50 K, green—250 K, light orange—255 K, orange—260 K, red—265 K, dark red—270 K). (**d**) illustrates the thickness of the QLL formed at different temperatures on the three basal planes of the AAA-1 asphalt on ice (dark green represents the basal plane, light green represents the primary prismatic face, and orange represents the secondary prismatic face).

It was hypothesized that the three crystal faces of ice would create anisotropic variations in the QLL thickness. Therefore, separate interface models were developed for the AAA-1 asphalt on each ice crystal face. Furthermore, the QLL thickness for various crystal faces was determined at different temperatures. Figure 7a–c show the variation in QLL thickness over time during the simulation for the three crystal faces, offering a clearer view of how QLL thickness evolves with temperature changes. As Figure 7d illustrates, at a temperature nearing the melting point of TIP4P-ICE (270 K), the QLL thickness on the basal plane reached a maximum of 23.91 Å, while it was at its minimum of 8.71 Å on the secondary prismatic face, and intermediate on the primary prismatic face. At any given temperature, the basal plane consistently presents the thickest layer, with its thickness increasing from 1.13 times to 2.92 times that of the secondary prismatic face as the temperature rises. The primary reason for these thickness differences is the variation in premelting initiation temperatures across different crystal faces. Simulations and data suggest that premelting on the basal plane begins around 170 K, compared to approximately 200 K on the primary prismatic face and 210 K on the secondary prismatic face. Since premelting starts earliest on the basal plane, increasing temperatures and extending time led to more pronounced premelting of ice on this face, resulting in the continual accumulation of water molecules and an increase in QLL thickness.

Moreover, the hexagonal structure of the basal plane leads to a higher surface energy compared to the other two faces. As this energy increases, the melting phenomenon becomes more pronounced near the melting point, accounting for the thickness increase to 2.92 times at this temperature. Additionally, the basal plane possesses the highest surface atom density, facilitating the formation of hydrogen bonds with heteroatoms in asphalt. These enhanced interactions contribute to accelerated premelting of ice, resulting in the greatest QLL thickness on the basal plane.

The basal plane is integral to the structure of ice crystals, being the first plane to initiate premelting and exhibiting the thickest QLL. In an actual icing pavement scenario, the interface between ice and asphalt primarily involves the basal plane. Investigating the QLL thickness at this interface through molecular dynamics can yield valuable insights into their adhesive properties, offering significant implications for future research.

#### 4.1.2. Impact of Temperature on the QLL Thickness

**Figure 8:**
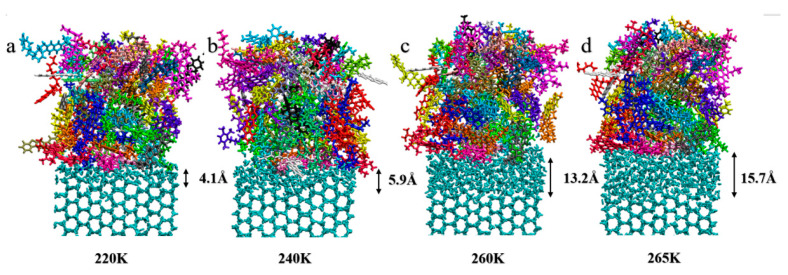
Observation of the thickness of the QLL at the AAA-1-asphalt–ice interface at different temperatures: (**a**–**d**) correspond to temperatures of 220 K, 240 K, 260 K, and 265 K, respectively.

To find out how temperature affects the thickness of the QLL, the QLL thickness was calculated at the interface between AAA-1 asphalt and the basal plane of ice crystals across temperatures ranging from 200 K to 270 K. Figure 8 demonstrates the interface simulation at four distinct temperatures. It becomes clear that as the temperature incrementally increases from the premelting point to the melting point, the QLL thickness consistently increases. After the premelting phase, the QLL was 5.93 Å thick at 220 K, increasing to 7.87 Å at 240 K, reaching 13.17 Å at 260 K, and further expanding to 15.77 Å at 270 K. Simultaneously, the QLL thickness measured through temperature is similar to the recent test result of 1.3 nm by Zhang [15]. Therefore, it is evident that temperature significantly impacts the QLL thickness.

In thermodynamics, as the temperature increases, it augments the thermal kinetic energy of molecules at the ice–asphalt interface, resulting in an expanded distance between molecules. Concurrently, the system becomes more disordered, contributing to a more chaotic QLL and consequently an increase in its thickness. A rise in temperature boosts the kinetic energy of molecules at the interface, thus accelerating their diffusion rates. Higher temperatures also enhance the likelihood of overlapping surface ice molecules, further affecting the QLL thickness. Figure 8b–d effectively illustrate the interaction of water molecules in the QLL with asphalt. An increase in temperature leads to more hydrogen bonds forming between the water molecules at the interface and the heteroatoms present. These interactions at the interface, intensifying with rising temperature, modify the surface tension and wetting properties, thereby impacting the QLL thickness. The importance of these interactions will be further highlighted in the subsequent discussions on adhesive properties.

**Figure 9:**
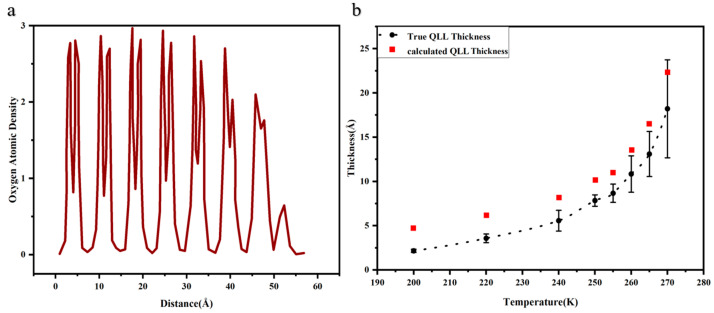
(**a**) Oxygen atom density diagram of AAA-1-asphalt–ice basal plane. (**b**) Estimated range for the true thickness of the QLL.

In the examination of the basal plane’s average density at 260 K, the cross-sectional density results are presented in Figure 9a. This figure conceptually delineates the number of layers formed in the liquid film. At lower temperatures, only the external water molecules contribute to the formation of the QLL. With the temperature rising, a dual-layer ice melting process becomes apparent. The basal plane’s density diagram exhibits two peaks, each representing three oxygen atoms within the hexagonal ring structure. Peaks displaying irregular patterns can be attributed to quasi-water molecules. As the graph does not precisely define the QLL thickness, an estimated range for its thickness is proposed. The observed trend is consistent with the tetrahedral order method, exhibiting only minor numerical discrepancies, thereby corroborating the estimated QLL thickness.

Significantly, upon reducing the temperature to 50 K for interface simulation, the snapshot indicates an absence of the QLL. Nonetheless, the thickness of the QLL, as determined through tetrahedral order, is nonzero. This inconsistency is attributed to the tetrahedral order parameter *q*, which considers the four nearest neighboring oxygen atoms. However, the surface’s outermost hydrogen atoms are incapable of forming a tetrahedron, leading to their misclassification as quasi-liquid. Therefore, we introduce a modified definition of the QLL thickness, detailed in Equation (19). Figure 9b depicts the adjusted range for the actual thickness of the QLL, omitting the influence of the surface’s outermost ice layer.

(19) $$\delta_{true}\left(T\right)=\delta_{apparent}\left(T\right)-\delta_{apparent}\left(T=50\;K\right)$$

#### 4.1.3. Impact of Different Types of Asphalt on the Thickness of the QLL

**Figure 10:**
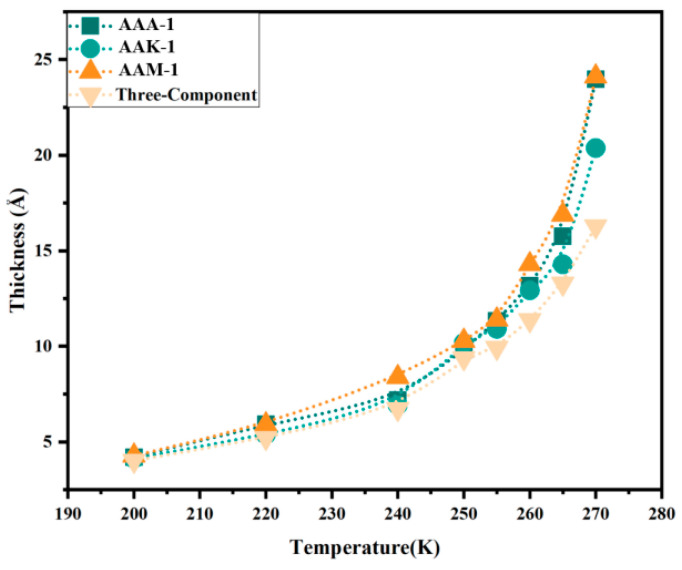
Thickness of the QLL formed at the interface of the basal plane of ice for four different asphalts.

The thickness of the QLL is primarily influenced by the properties of the ice crystal. However, when considering the asphalt–ice interface, the QLL’s thickness varies across different asphalt types. The QLL models were analyzed for four asphalt varieties in contact with ice, and the QLL thickness was measured at various temperatures. These findings are depicted in Figure 10. Notably, asphalt consisting of 12 components exhibits a significantly thicker QLL compared to asphalt with only 3 components. This difference is attributed to the composition of the three-component mixture, which comprises asphaltene, saturated hydrocarbons, and naphthene aromatics. Among these, saturated hydrocarbons and naphthene aromatics are low in polarity, with only asphaltene contributing to the mixture’s polarity, albeit in a smaller proportion. Non-polar molecules induce weaker interaction forces with water molecules at the ice interface, leading to a reduced transformation into liquid water. Moreover, the three-component mixture contains minimal oxygen, while the 12-component mixture is rich in oxygen, nitrogen, and sulfur atoms. These atoms, with their higher electronegativity, are more effective in attracting water molecules from the ice, particularly nitrogen and oxygen atoms, which are more likely to form hydrogen bonds. Consequently, the 12-component asphalt’s QLL is thicker than that of the 3-component variant.

However, at the ice interface, the QLL thickness varies among three 12-component asphalts: AAA-1, AAK-1, and AAM-1. Although the QLL thicknesses of these asphalts are similar, AAM-1 contains a significantly higher number of water molecules in its central part. This difference is not attributable to variations in asphaltene molecule numbers, as they remain consistent across all three asphalts. Instead, the variation stems from AAM-1’s higher concentration of polar aromatic compounds, such as Quinolinohopane, Thioisorenieratane, Trimethylbenzeneoxane, and Pyridinohopane, compared to AAA-1 and AAK-1. These compounds engage more effectively with water molecules on the ice surface, facilitating their transition into a quasi-liquid state and consequently increasing the QLL thickness. Additionally, the viscosity of AAM-1 asphalt at 220 K, measured at 283.15, is lower than that of the other two 12-component asphalts. A higher viscosity limits the mobility of water molecules at the interface; thus, an increase in asphalt viscosity correlates with a decrease in QLL thickness. Notably, AAA-1 contains a greater amount of Benzobisbenzothiophene, a small, polar molecule, compared to AAK-1. These molecules diffuse more rapidly at the interface, and their oxygen atoms are more prone to forming hydrogen bonds. Moreover, temperature also plays a role in influencing QLL thickness by affecting the interfacial tension and contact angle.

### 4.2. Adhesive Characteristics of Asphalt–QLL–Ice

#### 4.2.1. Interfacial Tension and Contact Angle of the Asphalt–QLL–Ice

**Figure 11:**
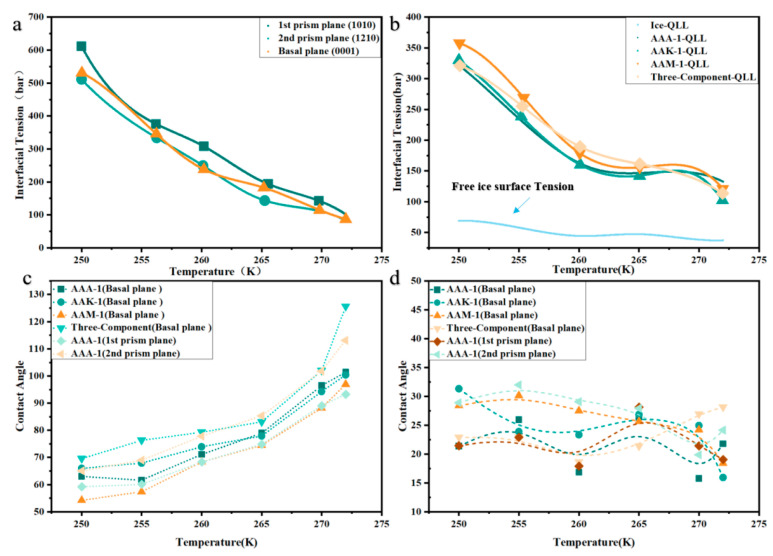
At a temperature of 220 K: (**a**) Interface diagram of AAA-1 asphalts with ice. (**b**) Interface tension of different asphalts. (**c**) Asphalt–QLL contact angle. (**d**) Ice–QLL contact angle.

In the study of interfacial dynamics between solid and liquid phases, the interfacial tension and contact angle are pivotal in assessing interactions and adhesion properties. Interfacial tension is a key descriptor of mechanical characteristics across different phases, quantifying the force per unit area. It can not only elucidate interfacial interactions but also establish a basis for calculating adhesion strength by analyzing interfacial tension between a liquid film and asphalt, and between the liquid film and ice. Figure 11a shows how interfacial tension varies for AAA-1 asphalt across three crystal faces at varying temperatures. Figure 11b illustrates interfacial tension profiles for four asphalt types at different temperatures and includes the interfacial tension curve between ice and the liquid film. It is evident that the interfacial tension at the ice–liquid film interface is substantially higher than at the asphalt interface, attributable to asphalt’s higher polarity and more irregular molecular surface, which favors lower interfacial tension with ice. Of the four asphalts analyzed, AAM-1 demonstrates the highest interfacial tension, likely due to its higher concentration of polar molecules like Quinolinohopane, Thioisorenieratane, Trimethylbenzeneoxane, and Pyridinohopane. Generally, interfacial tension decreases as temperature increases, owing to enhanced molecular thermal motion reducing interaction forces and interface energy. Concurrently, a drop in asphalt viscosity further lowers interfacial tension.

The contact angle between the condensed layer and the interfaces of ice and asphalt effectively illustrates the interactions and wettability. Figure 11c reveals the contact angles for the condensed layer on four asphalt types at various temperatures. For all asphalts, the trend with rising temperature is consistent. Below 265 K, contact angles remain under 90°. Above 270 K, they exceed 90°, indicating reduced wettability near the melting point, which correlates with weaker cohesion and adhesion. This suggests stronger attraction between the condensed layer’s water and the solid asphalt at lower temperatures, making ice removal more challenging. Figure 11d shows contact angle relationships for the condensed layer on ice in the four asphalt–ice models. The contact angles on ice are generally low, signaling stronger water–ice interactions. As temperature increases, emerging interactions between the water in the condensed layer and the ice compensate for temperature effects on the contact angle.

#### 4.2.2. Adhesive Characteristics of Asphalt–QLL–Ice Interface

The central focus of this investigation is the adhesion characteristics at the asphalt–ice interface. It is crucial to accurately calculate the adhesion strength for understanding how variations in interface parameters affect the QLL’s adhesion properties at this interface. The asphalt–QLL–ice system is conceptualized as a capillary adhesion model, wherein liquid bridges the gap between two solid, flat surfaces. In this model, the liquid generates additional capillary pressure, acting as an adhesive force between the solids. This additional pressure is analogous to the QLL interface’s adhesion strength that requires calculation.

**Figure 12:**
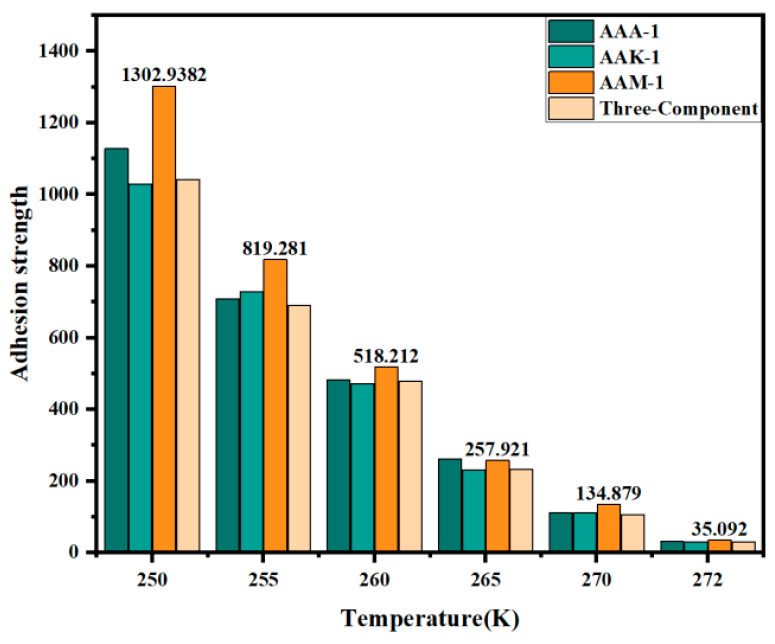
Adhesion strength (Pa) at the QLL interface for four types of asphalt under different temperature.

Previous studies have shown that key factors affecting adhesion strength include the QLL’s surface tension and thickness, as well as the contact angle θi of the QLL with both the ice and asphalt. These parameters are influenced by temperature changes and different asphalt types. Figure 12 illustrates the adhesion strength of ice interfaces with various asphalts under temperature gradients. A clear trend is observed: the adhesion strength decreases as the temperature approaches the ice’s melting point. At 250 K, the adhesion strength for all four asphalts exceeds 1 kPa, whereas it does not surpass 40 Pa near the melting point of 272 K. This decrease in adhesion strength can be linked to increased molecular motion and activity at higher temperatures, which weakens intermolecular forces and disrupts most hydrogen bonds between asphalt and ice. Additionally, a rise in temperature elevates the system’s entropy, reducing molecular orderliness and consequently diminishing interfacial adhesion strength. Further, water produced in the QLL can act as a lubricant between the asphalt and ice. This study indicates that temperatures closer to the melting point correlate with an increased thickness of the QLL. As the QLL’s thickness and the number of water molecules within it rise, the resulting enhanced lubrication effect leads to reduced adhesion strength.

**Figure 13:**
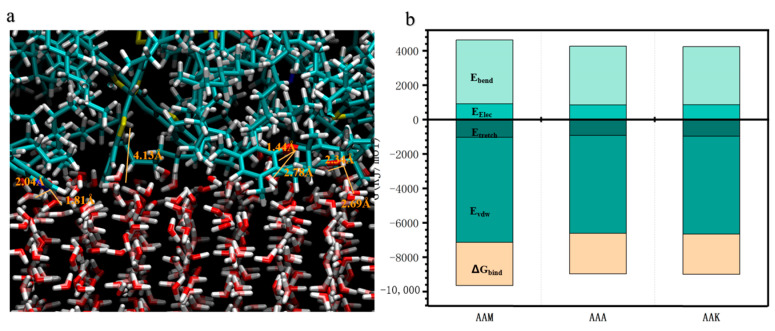
(**a**) Simulation details of the hydrogen bond formation between AAA-1 asphalt and the ice crystal surface at 220 K. (**b**) Average binding free energy and composition of the three asphalts after interface simulation.

Figure 12 further demonstrates the comparative adhesion strength across four asphalt types. Data reveal that at a constant 250 K, the adhesion strength of 12-component asphalt is ranked from highest to lowest as AAM-1, AAA-1, and AAK-1. Across the temperature range, AAM-1 consistently exhibits the highest adhesion strength. In this study, the numerical values of adhesive strength obtained are smaller compared to the results of molecular dynamics simulations of asphalt–aggregate, which is reasonable since the interactions between water and an aggregate with asphalt are also different. Detailed contact imagery of AAM-1 asphalts with the ice QLL, shown in Figure 13a, indicates a higher prevalence of polar aromatic hydrocarbon molecules at the interface compared to the other two types. The key factor in adhesion strength is the formation of hydrogen bonds between the asphaltene molecules and the polar aromatic hydrocarbons’ oxygen, nitrogen, and sulfur atoms and the QLL’s water molecules. Specifically, two oxygen atoms and one nitrogen atom are observed forming hydrogen bonds with the QLL water molecules, while sulfur atoms do not, likely due to their lower electronegativity and resultant weaker hydrogen bond formation.

In the investigation of the relationship between adhesion strength and the thickness of the QLL, it has been observed that as temperature increases, adhesion strength decreases. This temperature variation directly influences the thickness of the QLL. The capillary addition pressure formula previously derived suggests that a thicker QLL corresponds to lower adhesion strength. Molecular dynamics (MD) simulations demonstrate that an increase in QLL thickness leads to a higher number of free water molecules at the surface, which act as a lubricant. By calculating the number of hydrogen bonds at various temperatures and QLL thicknesses, it is evident that a thicker QLL layer has fewer hydrogen bonds. The reduction in the hydrogen bond quantity leads to an enhanced surface migration rate of water molecules. With the randomization of the molecular structure at the interfaces, it consequently diminishes interfacial friction, leading to a decrease in the adhesion strength of the QLL interface.

**Figure 14:**
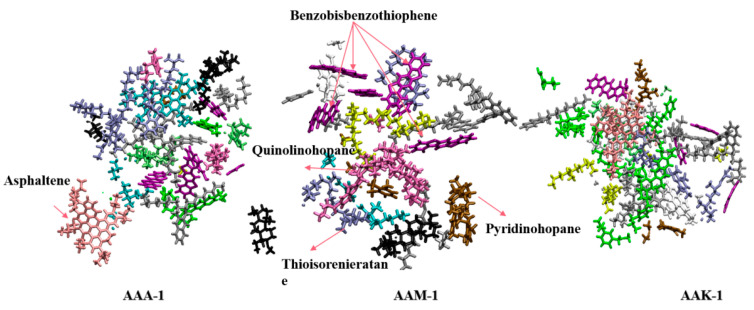
Cross-sectional diagrams of the contact points at the interface of three types of asphalt.

Figure 14 presents a cross-sectional view of the three asphalt types in contact with the QLL. This view distinctly shows the varying molecular species at each asphalt type’s QLL interface. AAM-1 has a notably higher presence of polar aromatic hydrocarbon molecules at its QLL contact surface compared to the others. This is attributed to AAM-1’s greater proportion of five types of aromatic hydrocarbons, enhancing its likelihood of interacting with water molecules on the QLL surface and leading to stronger polar interaction forces. Additionally, this composition increases its propensity to form stronger hydrogen bonds. For the other two asphalt types, the moderately polar asphaltenes also contribute to adhesion strength at the interface. Importantly, the aromatic hydrocarbon structures in asphalt engage with the ice surface’s electron cloud through π–π interactions, even before premelting forms the QLL. Therefore, an increase in polar aromatic hydrocarbons significantly boosts the asphalt–QLL interface’s adhesion strength, irrespective of premelting.

The binding free energy of three distinct asphalts at the ice interface at 250 K is illustrated in Figure 12b, after extracting the simulation trajectory. Given the minimal deformation of the asphalt molecules’ main chain at the interface, the contribution of Ebonded to the binding free energy was deemed inconsequential. Additionally, the effect of the system’s entropy on this energy was considered negligible. The computed binding free energies for AAA-1, AAM-1, and AAK-1 asphalt were −2349 kJ/mol, −2509 kJ/mol, and −2336 kJ/mol, respectively. These results highlight AAM-1 asphalt’s superior binding capability at the interface with the QLL. Notably, the proportion of Evdw was the most significant, underscoring its pivotal role in the interface’s binding stability. Moreover, a parallel is drawn between the binding free energy and adhesive strength trends: lower (more negative) binding free energy correlates with increased adhesive strength. This correlation emerges as binding free energy reflects the dynamics of intermolecular forces, which are crucial in influencing adhesive strength.

## 5. Conclusions

In this study, molecular dynamics (MD) simulations were utilized to model the molecular interactions at the interface between various asphalt types and ice. The research focused on assessing the impact of different asphalt compositions and temperature variations on the thickness of the quasi-liquid layer (QLL) and on the interfacial adhesion strength of these asphalts at diverse temperatures. The key findings are summarized as follows:

1. The QLL thickness varies among different asphalt types upon interaction with ice. At a constant temperature, the QLL thickness decreases in the order of AAM-1, AAA-1. As temperature increases, the system’s disorder grows, speeding up the surface melting and further thickening the QLL.
2. AAA-1 asphalt shows varying premelting temperatures on the three different crystal faces of ice, resulting in different QLL thicknesses. The premelting temperature on the basal plane is observed at 170 K, 200 K on the primary prismatic plane, and 210 K on the secondary prismatic plane. At any given temperature, the QLL thickness is highest on the basal plane, correlating with increased adhesion strength.
3. The adhesive strength at the asphalt–QLL–ice interface is influenced by the QLL thickness, the simulation temperature, and the QLL’s surface tension. There is an inverse relationship between adhesive strength and QLL thickness because of free water molecules acting as lubricants at the interface.
4. Of the studied asphalts, AAM-1 exhibits the highest adhesive strength at the QLL–ice interface, measured at 1306.93 Pa at 250 K. This is attributed to its high proportion of polar aromatic hydrocarbons. In cold regions, it is advisable to avoid selecting asphalts similar to AAM-1, which contains a high proportion of polar aromatic hydrocarbons, as road surface materials.

## Figures and Tables

**Table 1:** Fundamental information of Molecules in the AAA-1 Asphalt Model [22].

Molecule	Molecular Formula	Atom Mass Fraction (C_arom_)	Atom Mass Fraction (S)	Atom Mass Fraction (N)
Asphaltene-thiophene	C_51_H_62_S	0.42	0.000	0.000
Asphaltene-pyrrole	C_66_H_81_N	0.41	0.000	0.16
Asphaltene-phenol	C_42_H_50_O	0.41	0.045	0.000
Squalane	C_30_H_62_	0.00	0.000	0.000
Hopane	C_29_H_50_	0.00	0.000	0.000
PHPN (perhydrophe-nanthrene-naphthalene)	C_35_H_44_	0.41	0.000	0.000
DOCHN (dioctyl-cyclohexane-naph-thalene)	C_30_H_46_	0.30	0.000	0.000
Quinolinohopane	C_34_H_47_N	0.20	0.000	0.025
Thioisorenieratane	C_40_H_60_S	0.34	0.056	0.000
Trimethylbenzeneoxane	C_29_H_50_O	0.17	0.000	0.000
Pyridinohopane	C_30_H_45_N	0.12	0.000	0.028
Benzobisbenzothiophene	C_18_H_10_S2	0.74	0.22	0.000

**Table 2:** Four asphalt models containing twelve-component numbers.

Molecule	AAA-1	AAK-1	AAM-1	Three-Component	Molecule
Asphaltene-thiophene	3	3	3	27	Dimethylnaphthalene
Asphaltene-pyrrole	2	2	2	5	Asphaltene
Asphaltene-phenol	3	3	3	41	Docosane
Squalane	4	2	1		
Hopane	4	2	1		
PHPN	11	10	20		
DOCHN	13	10	21		
Quinolinohopane	4	4	10		
Thioisorenieratane	4	4	10		
Trimethylbenzeneoxane	5	5	10		
Pyridinohopane	4	4	10		
Benzobisbenzothiophene	15	12	4		

**Table 3:** Basic parameters of the TIP4P-ICE water model.

	θ/k _(K)_	α _(Å)_	q _(H) (e)_	q _(O) (e)_	q _(M)(e)_	r_OM (Å)_	r_OH (Å)_	∠HOH _(o)_
TIP4P-ICE	106.1	3.1668	0.5897	0	−1.1794	0.15	0.9572	104.52

**Table 4:** Simulated asphalt property values compared with experimental measurements [28].

	Density at 272 K (g/cm^3^)	Glass Transition Temperature (K)	Viscosity at 372 K (cP)	CED/ (J/m^3^)
Calculation (12-AAA-1)	1.01	249.52	1.72	3.310 × 10^8^
Calculation (12-AAA-M)	1.03	276.77	1.81	3.490 × 10^8^
Calculation (12-AAA-K)	0.98	271.91	1.51	3.370 × 10^8^
Calculation (3)	0.95	283.19	1.42	3.420 × 10^8^
Experiment	0.95–1.04	250–400	1.4–4.0	2.34 × 10^8^–5.29 × 10^8^

## Data Availability

The data presented in this study are available on request from the corresponding author.
